# A System Based on the Internet of Things for Real-Time Particle Monitoring in Buildings

**DOI:** 10.3390/ijerph15040821

**Published:** 2018-04-21

**Authors:** Gonçalo Marques, Cristina Roque Ferreira, Rui Pitarma

**Affiliations:** 1 Unit for Inland Development, Polytechnic Institute of Guarda, Avenida Doutor Francisco Sá Carneiro N° 50, 6300-559 Guarda, Portugal; goncalosantosmarques@gmail.com; 2 Department of Imagiology, Hospital Centre and University of Coimbra (CHUC), 3000-075 Coimbra, Portugal; cris.rcf@gmail.com

**Keywords:** indoor air quality (IAQ), healthy buildings, occupational health, particulate matter (PM), real-time monitoring, internet of things (IoT)

## Abstract

Occupational health can be strongly influenced by the indoor environment as people spend 90% of their time indoors. Although indoor air quality (IAQ) is not typically monitored, IAQ parameters could be in many instances very different from those defined as healthy values. Particulate matter (PM), a complex mixture of solid and liquid particles of organic and inorganic substances suspended in the air, is considered the pollutant that affects more people. The most health-damaging particles are the ≤PM_10_ (diameter of 10 microns or less), which can penetrate and lodge deep inside the lungs, contributing to the risk of developing cardiovascular and respiratory diseases, as well as of lung cancer. This paper presents an Internet of Things (IoT) system for real-time PM monitoring named iDust. This system is based on a WEMOS D1 mini microcontroller and a PMS5003 PM sensor that incorporates scattering principle to measure the value of particles suspended in the air (PM_10_, PM_2.5_, and PM_1.0_). Through a Web dashboard for data visualization and remote notifications, the building manager can plan interventions for enhanced IAQ and ambient assisted living (AAL). Compared to other solutions the iDust is based on open-source technologies, providing a total Wi-Fi system, with several advantages such as its modularity, scalability, low cost, and easy installation. The results obtained are very promising, representing a meaningful tool on the contribution to IAQ and occupational health.

## 1. Introduction

People spend about 90% of their time in indoor environments, and especially the elderly population and newborns, who are the most susceptible to indoor pollution, may spend all their time in indoor environments. Thus, indoor air quality (IAQ) is the main descriptor of personal exposure to pollutants [1].

Poor IAQ is assumed as a challenge of greatest significance which particularly affects the poorest and unprotected individuals worldwide who are most defenseless introducing itself as a significant issue for world well-being, that can be compared with others such as tobacco use or sexually transmitted diseases [2]. The U.S. Environmental Protection Agency (EPA), responsible for indoor and outdoor air quality regulation in the USA, considers that indoor levels of pollutants might be up to 100 times higher than outdoor air pollutant levels and positioned poor air quality as one of the most important five environmental problems to the general well-being [3].

In 2050, 20% of the world’s population will be age 60 or above [4], which will result in an increase in diseases, health care costs, shortage of caretakers dependency and brutal social impact. In fact, 87% of the people prefer to stay in their homes and bear the remarkable cost of nursing care [5], which makes the study of ambient assisted living (AAL) architectures undoubtedly a topic of great relevance given the aging of the world population. AAL technologies are designed to meet the needs of the aging population to maintain their independence as long as possible.

On the one hand, enhancements in networks, sensors, and embedded systems have made possible the provision of real-time monitoring and personalized healthcare systems to individuals that they can now use in their living environments. In another hand, these uninterrupted technological improvements make possible the development of intelligent cyber-physical systems for enhanced living environments and occupational health. Although there is a lot of challenges in the design and implementation of an effective AAL system such as information architecture, interaction design, human-computer interaction, ergonomics, usability and accessibility [6], there are also social and ethical problems like the acceptance by the older adults and the privacy and confidentiality that should be a requirement of all AAL devices. In fact, it is also important to ensure that technology does not replace the human care but rather should be a remarkable compliment.

IoT is a paradigm where objects are connected to the internet and support sensing capabilities. Tendentiously IoT devices should be ubiquitous, context-aware and enable ambient intelligence features closely related to AAL. IoT is a suitable approach to build health care systems; technology advancements allow one to define new advanced tools and also allow real-time health monitoring platforms for decision making in the treatment of many diseases. IoT offers a perfect platform for ubiquitous healthcare, using, for example, wearable sensors for uploading data to servers and smartphones for communication, along with Bluetooth for interfacing sensors measuring physiological parameters [7]. The IoT is also used for real-time transmission of physical information. In 2009 some projects for remote health care program design based on IoT that can improve the intelligence of information collection and process already existed, which promoted IoT applications in the medical industry [8]. Despite the potential of the IoT paradigm and technologies for health systems today many challenges to be overcome still exist. The direction and impact of the IoT on the economy are not yet clear, there are barriers to the immediate and ubiquitous adoption of IoT products, services, and solutions which may sound feasible for implementation, but the timing might be too early [9].

An IAQ evaluation system helps in the monitoring and decision making at real-time interventions to increase occupational health. Local and distributed assessment of chemical concentrations is significant for safety (e.g., gas spills detection and pollution monitoring) and security applications, as well as to control heating, ventilation, and air conditioning (HVAC) systems for energy efficiency [10]. In fact, the IAQ measured in the manufactured environment provides a consistent stream of data for reliable management of building automation systems and provides a platform for informed decision-making [11].

In most situations, easy interventions that can be carried out by homeowners and building administrators can deliver remarkable positive effects on IAQ, for example, the avoidance of smoking inside and the utilization of natural ventilation are critical practices that should be instructed to families through instructive projects that address the IAQ [12].

Numerous IoT architectures for IAQ supervision which support open-source technologies for data processing, collection, and transmission that offers mobile computing architectures for real-time data accessibility are proposed in [13,14,15,16,17,18,19,20,21,22]. Particularly, IAQ monitoring is a trending topic for which some other low-cost and open-source monitoring systems that had been developed [23,24,25,26,27]. A brief summary of these studies is presented in Table 1.

This paper aims to present iDust, a solution for real-time monitoring based on an IoT architecture. To create a low-cost system for PM, the authors selected a PMS5003 sensor that incorporates scattering principle to measure the value of particles suspended in the air with a diameter of 10 microns or less (≤PM_10_), 2.5 microns or less (≤PM_2.5_) and 1.0 microns or less (≤PM_1.0_).

The paper is structured as follows: after this Introduction (Section 1), Section 2 introduces in detail the PM exposure effects in health; Section 3 is concerned to the methods and materials used in the implementation of the sensor system; Section 4 demonstrates the system operation and experimental results; Finally, the conclusions are presented in Section 5.

## 2. Particulate Matter’s Effects on Health

PM can be defined as a multifaceted combination of solid and liquefied biological and mineral material particles suspended in the air and is therefore mentioned by several researchers as the pollutant that influences more individuals [28,29]. PM include dust, dirt, soot, smoke, and liquid droplets that are capable of penetrating the lower airways of humans and can cause possible negative health effects. In developing countries, indoor exposure to PM increases the risk of acute lower respiratory infections and associated mortality among young children, but also represent a major risk factor for cardiovascular disease, chronic obstructive pulmonary disease and lung cancer among adults. Typically there are strong similarities between airborne particulate matter sampled in cities in developed countries across the world [30].

Already in 1987, the US EPA redefined the National Ambient Air Quality Standard (NAAQS) for particles based on particulate matter smaller than PM_10_ [31]. Environmental tobacco smoke is a major contributor to indoor air concentrations and human exposures to particles [32]. The World Health Organization (WHO) considers as threshold values for PM_10_ and PM_2.5_ 20 and 10 μg·m^−3^ (annual mean) or 25 and 50 μg·m^−3^ (24-h mean), respectively [33]. There is increasing evidence of PM-related cardiovascular health effects, as well as a pathophysiological interconnection that links PM exposure with cardiopulmonary morbidity and mortality [34,35].

**Figure 1:**
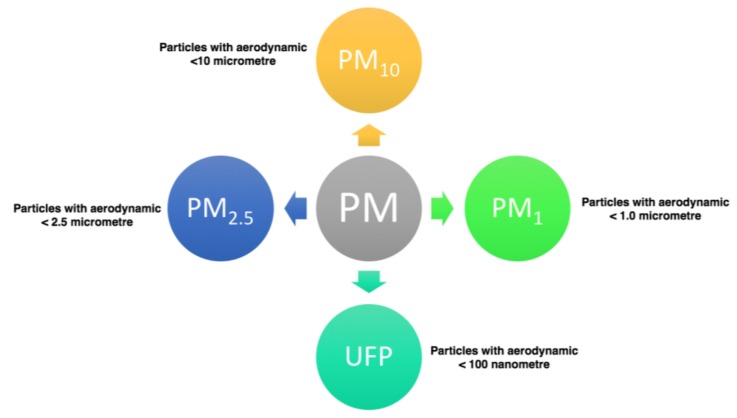
PM types.

For routine monitoring for regulatory purposes, ambient PM is quantified via PM_10_ and PM_2.5_ metrics. PM_10_ and PM_2.5_ are defined as the mass concentrations of particles within a size fraction collected by automatic systems with inlet transmission curves that respect international sampling standards related to ‘inhalable’ and ‘respirable’ particles, respectively. Generally, PM_10_ (particles equal to and less than 10 microns in aerodynamic diameter) are not deposited in the lungs. PM_10–2.5_ (commonly defined as particles with an aerodynamic diameter greater than 2.5 microns, but equal to or less than a nominal 10 microns) are also known as coarse fraction particles. Particles smaller than 1 micron in diameter are represented by PM_1.0_. “Ultrafine” particles (UFP) are generally defined as the particles less than 0.1 microns (100 nm) in size (Figure 1) [36].

Today the UFP concentrations are not monitored routinely, and enhanced measurement is necessary as it would support the development of more powerful epidemiological studies [37,38].

PM_2.5_ is associated with all-cause, lung cancer, and cardiopulmonary mortality and each 10 µg·m^3^ elevation in PM_2.5_ was associated with approximately a 4%, 6%, and 8% increased risk of all-cause, cardiopulmonary and lung cancer mortality, respectively [39].

Epidemiological studies point to a consistent association between particulate air pollution and not only exacerbations of illness in people with respiratory disease, but also rises in the number of deaths from cardiovascular and respiratory disease among older people [40]. As referred by [41] particle pollutants are estimated to cause more than 500,000 deaths annually, and the adverse effects of ultrafine air particles are linked to their ability to gain access to the lung and systemic circulation, where toxic components lead to tissue damage and inflammation. PM measurements can be intensely predisposed to mechanically produced, soil-derived particles, which could not be linked with unfavorable health effects so IAQ monitoring should include PM concentration [42].

The allergist–clinical immunologist should be strongly aware that both gaseous and particulate outdoor pollutants might augment or improve the fundamental pathophysiology of both the upper and lower airways [43].

Observed health effects of PM include increased respiratory symptoms, decreased lung function, increased hospitalizations, other healthcare visits for respiratory and cardiovascular disease, increased respiratory morbidity as measured by absenteeism from work and school or other restrictions in activity and increased cardiopulmonary disease mortality as referred by [44]. Through iDust, the medical decision and diagnosis can be supported by historical environment’s data of PM exposure where the patient lives serving as a powerful tool for real-time healthcare monitoring and decision making. A study suggesting that the acidity of particles is not as important in associations with daily mortality as the mass concentrations of particles is presented by [45]. Consequently the iDust focuses on the real-time monitoring of the concentrations of particles for enhanced occupational health.

PM exposure is ubiquitous and while there is no defined and studied “safe” level, behavioral modification strategies may contribute to better overall health. It’s necessary to enable policymakers, after weighing the economic impact, to establish enhanced legislation that limits PM exposure. There are natural PM sources such as volcanoes, forest fires that are unavoidable. However, some others sources are avoidable and several practices that can be administered keeping in mind the end goal to diminish PM exposure and decrease the morbidity and mortality [46].

In indoor environments, the health effects of inhaled biological particles can be significant, as a large variety of biological materials are present. One of the primary constituents of the smoke is respirable particles. These are aerosols with a small enough diameter to enter and remain in the lung, many are around 6–7 μm, or less in diameter [12]. Research that uses a sample of personalities conducted by [47,48] concludes that in general, the PM exposure in outdoor environments is greater than in indoor living environments, particularly in the daytime. Usually, the PM well-being effects of outdoor environments are reasonably well considered than those related to indoor living environments [49].

Tobacco smoke particles measure from 0.1 to 1.5 μm [50]. Research conducted by [51] concludes that a smoking one pack of cigarettes a day causes approximately 20 μg·m^−3^ to 24 h indoor concentrations and that short-term concentration of 500–1000 μg·m^−3^ are probable once the tobacco is lit. Several chronic exposure research studies suggest relatively general vulnerability to aggregated consequences of long-term PM_2.5_ exposure and consequential population average loss of life expectancy in extremely contaminated environments [52].

## 3. Materials and Methods 

**Figure 2:**
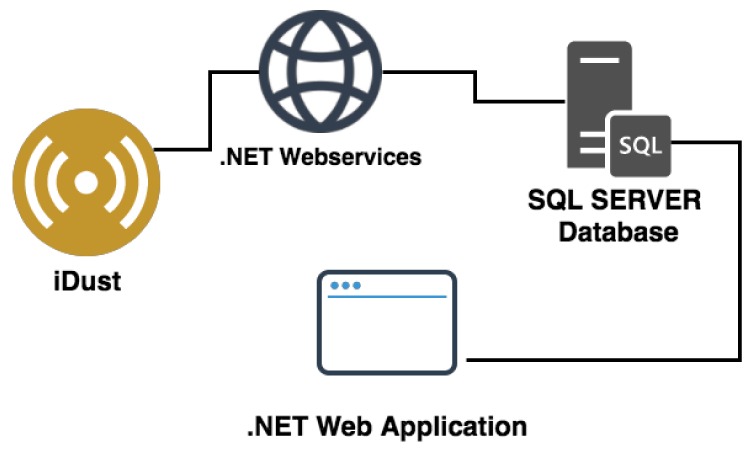
iDust system architecture.

Taking into account all the health negative effects of PM exposure described above, the authors had the objective of developing an automatic PM monitoring system called iDust. This system is intended to be a healthcare real-time monitoring system and decision-making tool. The solution is composed of a hardware prototype for ambient data collection and a Web portal developer in .NET for data consulting. The iDust is based on open-source technologies and is a totally Wi-Fi system, with several advantages compared to existing systems, such as its modularity, scalability, low-cost and easy installation. The data is uploading to the SQL SERVER database using .NET Web services (Figure 2). This system is based on a WEMOS D1 mini microcontroller with built-in Wi-Fi communication technology as communication and processing unit and incorporates a PMS5003 sensor as sensing unit.

**Figure 3:**
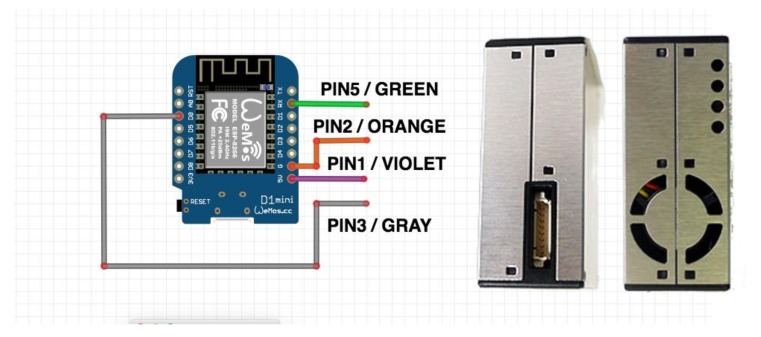
iDust Connection Diagram.

The iDust is a software/hardware solution for real-time IAQ supervising that acknowledges the end user, as the building supervisor for example, to real-time monitoring the PM exposure. The solution is based on the Arduino platform using the ARDUINO IDE for software development and the WEMOS D1 mini as a microcontroller. The parameters are monitored using the iDust prototype, which collects data that is stored in a SQL SERVER database using Web services developed in .NET. The end user can access the data from the Web portal developed using ASP.NET. After the user authentication, it is possible to access the real-time PM exposure data. The data collected is presented in a dashboard in both numeric values or chart form. Furthermore, the Web Application is responsible for saving the history of the PM exposure data. By offering a chronological history of the PM exposure data, this solution assists the builder manager to analyze the IAQ behavior accurately. This functionality is exceptionally significant on behalf of determining on conceivable interventions to increase the IAQ in the building. Additionally, this feature might stand particularly significant for therapeutic assessment and diagnosis support since the clinical team might analyze the environment where the patient resides. The Web Application remains additionally prepared with a powerful notification manager that alerts the user when a specific parameter outdoes the maximum rate. iDust is a low-cost, reliable system that can be easily configured and installed by the average user. For this, we selected a low cost but very reliable PM sensor and a microcontroller with native Wi-Fi support. This system consists of two components: a WEMOS D1 mini microcontroller (WEMOS Electronics, China) and a PMS5003 PM sensor (Plantower, Beijing, China), which incorporates scattering principle to measure the value of particles suspended in the air with a diameter of 10 microns or less (≤PM_10_), 2.5 microns or less (≤PM_2.5_) and 1.0 microns or less (≤PM_1.0_) (Figure 3).

A brief introduction of each component used is shown below

- WEMOS D1—This device incorporates an ESP8266 Wi-Fi chip with integrated antenna switches, RF balun, power amplifier, low noise receive amplifier, filters, and power management modules. It supports 802.11 b/g/n protocols, Wi-Fi 2.4 GHz, support WPA/WPA2, has an integrated low power 32-bit MCU, an integrated 10-bit ADC, has a standby power consumption of <1.0 mW (DTIM3) and can operate at temperature range −40~125 °C. The WEMOS D1 that offers 11/1 digital input/output pins, 1 analogue input, and a micro USB interface for development and power supply. It is totally supported by Arduino IDE platform, has an 80/160 MHZ CPU clock, 4 MB Flash, a 3.3 V operating voltage, a compact dimension of 34.2 mm × 25.6 mm, and a weight of 10 g.
- PMS 5003—this PM sensor was developed with special attention to its mechanical design which prevents dust accumulation where the laser and diode are mounted. Regarding electrical and electronic components, the PMS5003 incorporates Cypress CY8C4245 CPU, an ARM Cortex-M0 running at 48 Mhz with dedicated ADC. The PMS5003 applies the scattering principle to measure the value of particles suspended in the air with a diameter of 10 microns or less (≤PM_10_), 2.5 microns or less (≤PM_2.5_) and 1.0 microns or less (≤PM_1.0_). This PM sensor has a standby power consumption of <0.2 mW and can operate in a temperature range of −10~60 °C.

## 4. Results and Discussion

In Portugal, most buildings have natural ventilation. The type of dwelling, construction, heating, and ventilation all have a bearing on the extent to which air permeability changes. It is estimated that two-thirds of commercial/services buildings, with natural ventilation, are extremely airtight, and the remaining third tend to be leakier.

**Figure 4:**
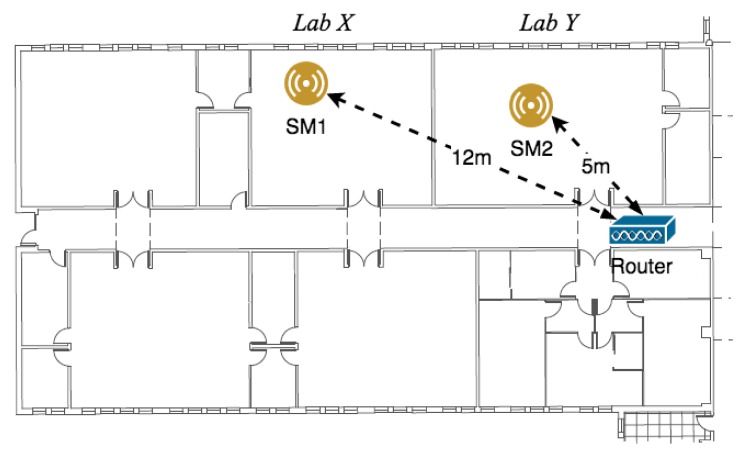
iDust installation schema.

For testing purposes, two laboratories of a Portuguese university were on-site monitored, and two iDust sensors modules (SM1 and SM2) were used. Figure 4 represents the experiment done by the authors of the implementation of the iDust system. As in the most buildings, the two spaces monitored are naturally ventilated, without any dedicated ventilation slots on the facades. The indoor air is heated and recirculated by two typical air-water fan-coils from the heating system, and the air exchange is achieved through infiltrations and opening windows controlled by the occupants. Once the outdoor air is only used to provide ventilation or to cool normally when the occupants feel the bad or irritating smell, the IAQ is normally poor.

All modules are powered by the power grid using a 230V-5V AC-DC 2A power supply. PM exposure data were collected for two months which showed that under certain conditions air quality values are significantly lower than those considered healthy for standards. The tests conducted show the system capability to analyze in real-time the IAQ, the potential to planning interventions to ensure safe healthy and comfortable conditions, but also to identify multiple situations or habits that affect the IAQ negatively.

**Figure 5:**
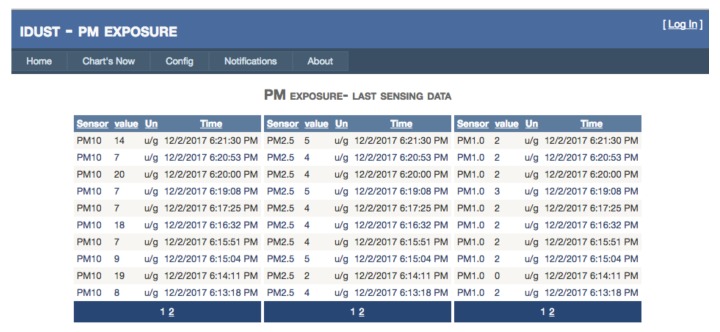
iDust Web application.

Using the Web application, the end user can easily access the PM exposure data in real time. The Web Application lets the end-user to keep the parameters history as is showed in Figure 5. This functionally provides precise and detailed access to the PM exposure behavior.

**Figure 6:**
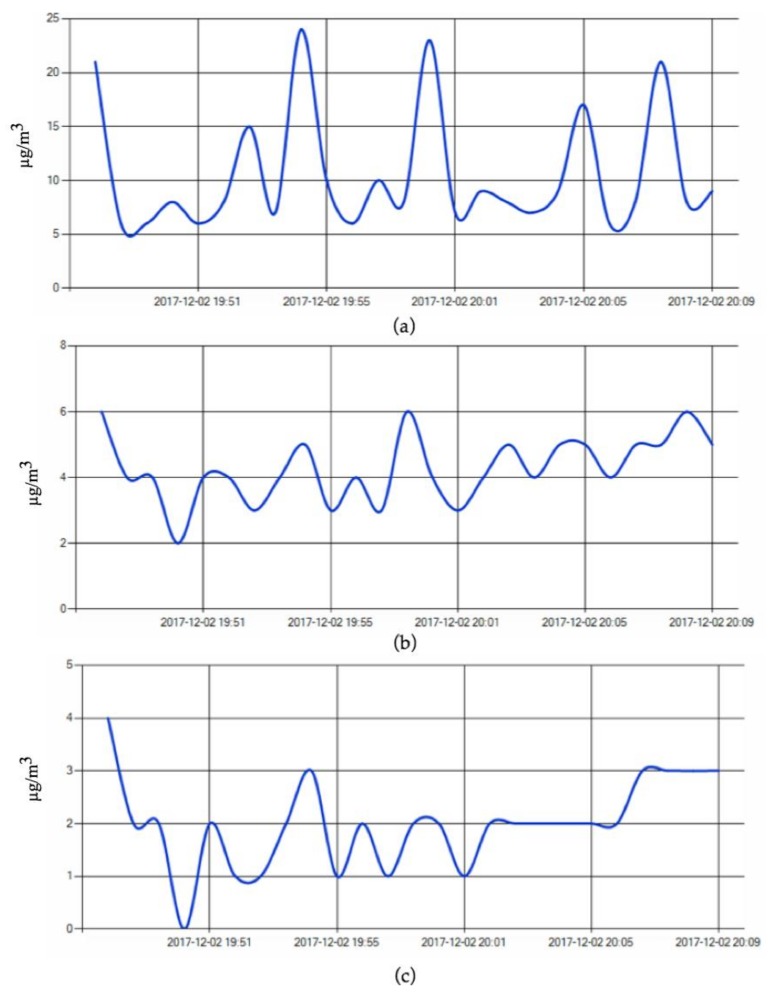
Results of particulate matter concentrations obtained in the experiments conducted in a real environment: (**a**) PM_10_ (µg/m^3^); (**b**) PM_2.5_ (µg/m^3^); (**c**) PM_1.0_ (µg/m^3^).

A sample of the graphics with the results obtained in the experiments collected by iDust is shown below (Figure 6). It should be noted that the graphs displayed the results obtained in the monitored rooms with induced simulations using tobacco smoke.

**Figure 7:**
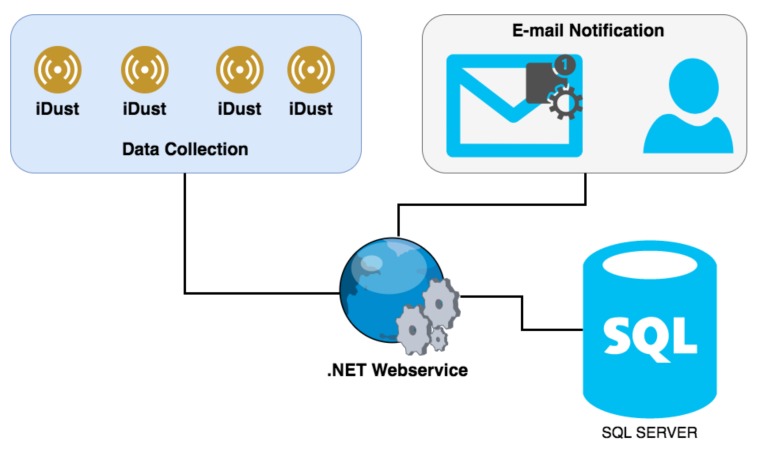
iDust Notification Architecture.

The iDust is also equipped with a powerful alerts manager that notifies the user when the air quality is poor. Based on well-studied values, the maximum and minimum health quality values are predefined by the system, but the user can also change this values to specific proposes. When a value exceeds the defined threshold, the user will be notified by e-mail in real time. Other notifications are planned as future work such as Short Message System (SMS, Figure 7).

The data collected by the system is analyzed before being inserted into the database if the data exceeds the parameterized limits the user receives are notified, and the email is triggered. Consecutively the user can act in real time ensuring good ventilation of the indoor environment.

The graphical display of the air quality data allows a greater perception regarding the behavior of the monitored parameters than the numerical display format. On the other hand, the Web application also allows a more precise analysis of the detailed temporal evolution. Thus, the system is a powerful tool for analyzing air quality consumption and to support decision making on possible interventions to improve a healthy and more productive indoor environment.

The iDust has advantages both in ease of installation and configuration due to the use of wireless technology but also due to its small size (about 5.5 cm × 4.5 cm × 4.5 cm depth), compared to other systems and also have a Web Application to provide relevant information anytime and anywhere to the users.

Compared to similar solutions the iDust provides a web platform for data consulting and notifications. It is relevant to support the planning of interventions on the building and to be shared with a medical team indoor to support diagnostics. In fact, we spend about 90% of our lives in indoor environments, so it is necessary to monitor the PM level to plan changes of habits and even interventions in the ventilation to provide a healthier and productive living environment. This system makes a significant contribution compared to existing air quality monitoring systems due to its low cost of construction, installation, modularity, scalability and easy access to monitoring data in real time through the web application.

**Figure 8:**
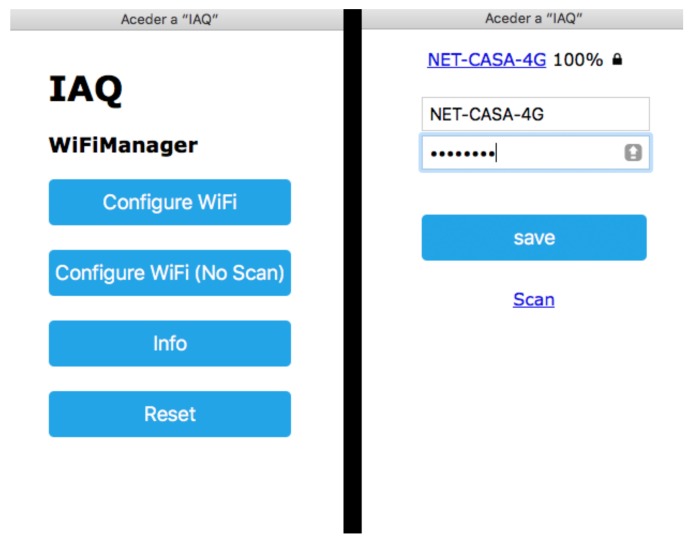
iDust Wi-Fi configuration.

The iDust system uses the ESP8266 for both processing and Internet connectivity, which offers several advantages regarding reducing the cost of the system, but also improves processing power because the ESP8266 has an 80 MHZ CPU, while the Arduino UNO has a 16 MHZ CPU. The use of the ESP8266 has another important feature that provides to the end user an easy configuration of the Wi-Fi network to which iDust will be connected. The ESP8266 is by default a Wi-Fi client, but in the case, it is unable to connect to the Wi-Fi network, or if there are no wireless networks available, the ESP8266 will turn to hotspot mode and will create a Wi-Fi network with an SSID “IAQ-iDust.” At this point, the end-user can connect to this Wi-Fi network which permits the configuration of the Wi-Fi network to which the iDust is going to connect through the introduction of the network SSID and password using a web form (Figure 8).

These features make the iDust an easy to install product which follows the original paradigm of IoT solutions. In contrast of the majority of the IAQ monitoring solutions that must be installed by specialized professionals, the iDust can be configured by the end user requiring only a smartphone or another gadget with Wi-Fi connectivity, thus contributing to the low-cost aim of the iDust solution. Another great advantage of this system is the notification system that allows users to act in real time to significantly improve indoor air quality through the ventilation or deactivation of pollutant equipment.

Finally, the IoT architecture enables the scalability of the system providing flexibility and expandability as the user can start with a few stations for data collection, and add more stations as the needs and complexity of the indoor environment.

Improvements to the system hardware and software are planned to make it much more appropriate for specific purposes such as hospitals, schools, and offices. The system has advantages both in installation and configuration, due to the use of wireless technology for communications, but also because it was developed for to be compatible with all domestic house devices and not only for smart houses or high-tech houses.

**Figure 9:**
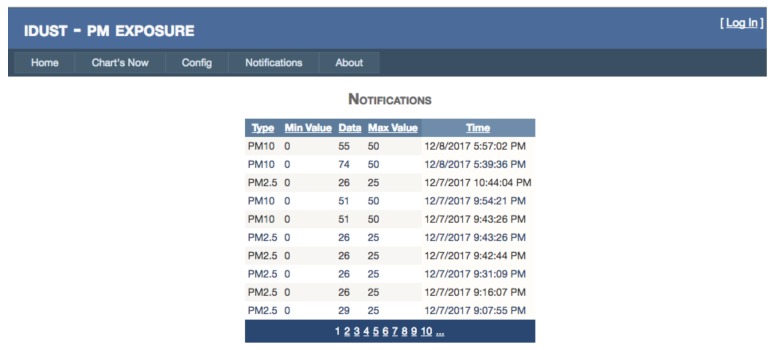
iDust Notifications.

The iDust not only provide a detailed PM exposure data in a FIGURE or graphic form but also provide real-time notifications for acting in real time (Figure 9).

It is imperative to control the PM exposure effectively and we believe that the first step is to perform real-time monitoring to perceive its variation in real-time and to plan interventions for its reduction.

IoT architectures and AAL technologies not only will remain side by side adding scientific improvements to enhanced living environments but also lower the cost of ubiquitous solutions. Despite the many technologic advances some difficulties in the construction of IoT solutions, several issues remain, mostly related to the privacy, data confidentiality, and security of such systems. Despite all the advantages of healthcare systems based on IoT architectures, several open issues continue to exist, such as availability, reliability, mobility, performance, scalability, and interoperability. Any proposed system should find ways to respond to these problems. It is extremely important to repeat that this kind of healthcare system should be used to support medical treatments as an important complement to medical supervision.

## 5. Conclusions

PM is related to numerous serious health problems. This paper presented iDust, a real-time PM exposure monitoring system and decision-making tool for enhanced healthcare based on an IoT architecture It was developed using open-source technologies and low-cost sensors. The results obtained are very promising, as the solution could be used to support the building manager for the appropriate operation and maintenance to deliver not just a safe but also a healthful workplace for enhanced occupational health. In the one hand, the PM exposure information can be particularly valuable to offer support to a medical examination by clinical professionals as the medical team might analyze the history of IAQ parameters of the ecosystem everywhere the patient lives and relate this records with his health complications. On the other hand, by monitoring IAQ, it is possible to perceive correctly the air quality conditions and if necessary plan interventions to decrease the PM exposure levels.

The results of the experiments reveal that iDust system can provide an effective PM assessment to prevent exposure risk. In fact, the IAQ may be extremely different than what is expected of a quality living environment. Therefore, this system is a useful tool for monitoring the PM of the indoor air and aims to ensure the permanent categorization of PM. It was also possible to conclude that the occupants only perceive the poor conditions of air quality in extreme situations, so by using iDust, it is possible to detect and correct the problems at a very early stage.

As future work, the authors plan software and hardware improvements to adapt the system to specific cases such as hospitals, schools, and industry. It is also important to create new monitoring solutions with the objective to develop an ecosystem for IAQ as well as the enhancements in the Web Application to increase the data safety and privacy for accessibility to health professionals planning to support medical diagnostics. We believe that in the future, systems like this will contribute to enhanced living environments but also be an integral part of the daily human routine.

## Figures and Tables

**Table 1:** A short summary of similar type of research on IoT platform for real-time indoor air quality monitoring.

	MCU	Sensors	Architecture	Low Cost	Open-Source	Connectivity	Data Access	Easy Installation
D. Lohani and D. Acharya [23]	Arduino UNO, ESP8266	Temperature, Relative Humidity, CO_2_	IoT	√	√	Wi-Fi, BLE	Mobile	×
P. Srivatsa and A. Pandhare [24]	Raspberry Pi	CO_2_	WSN/IoT	√	√	Wi-Fi	Web	×
F. Salamone et al. [25]	Arduino UNO	CO_2_	WSN	√	√	ZigBee	×	×
S. Bhattacharya et al. [26]	Waspmote	CO, CO_2_, PM, Temperature, Relative Humidity	WSN	×	√	ZigBee	Desktop	×
F. Salamone et al. [27]	Arduino UNO	Temperature, Relative Humidity, CO_2,_ Ligth, Air velocity	IoT	√	√	ZigBee/BLE	Mobile	×

MCU: microcontroller; √: apply; ×: not apply.
